# Functional and structural alterations of dorsal attention network in preclinical and early‐stage Alzheimer's disease

**DOI:** 10.1111/cns.14092

**Published:** 2023-03-21

**Authors:** Huimin Wu, Yu Song, Xinyi Yang, Shanshan Chen, Honglin Ge, Zheng Yan, Wenzhang Qi, Qianqian Yuan, Xuhong Liang, Xingjian Lin, Jiu Chen

**Affiliations:** ^1^ Department of Neurology The Affiliated Brain Hospital of Nanjing Medical University Nanjing China; ^2^ Institute of Neuropsychiatry The Affiliated Brain Hospital of Nanjing Medical University Nanjing China; ^3^ Institute of Brain Functional Imaging Nanjing Medical University Nanjing China; ^4^ Department of Radiology The Affiliated Brain Hospital of Nanjing Medical University Nanjing China; ^5^ Department of Radiology, Affiliated Drum Tower Hospital Medical School of Nanjing University Nanjing China; ^6^ Institute of Medical Imaging and Artificial Intelligence Nanjing University Nanjing China; ^7^ Medical Imaging Center, Affiliated Drum Tower Hospital Medical School of Nanjing University Nanjing China

**Keywords:** amnestic mild cognitive impairment, dorsal attention network, resting‐state functional magnetic resonance imaging, structural magnetic resonance imaging, subjective cognitive decline

## Abstract

**Objectives:**

Subjective cognitive decline (SCD) and amnestic mild cognitive impairment (aMCI) are known as the preclinical and early stage of Alzheimer's disease (AD). The dorsal attention network (DAN) is mainly responsible for the “top‐down” attention process. However, previous studies mainly focused on single functional modality and limited structure. This study aimed to investigate the multimodal alterations of DAN in SCD and aMCI to assess their diagnostic value in preclinical and early‐stage AD.

**Methods:**

Resting‐state functional magnetic resonance imaging (MRI) was carried out to measure the fractional amplitude of low‐frequency fluctuation (fALFF), regional homogeneity (ReHo), and functional connectivity (FC). Structural MRI was used to calculate the gray matter volume (GMV) and cortical thickness. Moreover, receiver‐operating characteristic (ROC) analysis was used to distinguish these alterations in SCD and aMCI.

**Results:**

The SCD and aMCI groups showed both decreased ReHo in the right middle temporal gyrus (MTG) and decreased GMV compared to healthy controls (HCs). Especially in the SCD group, there were increased fALFF and increased ReHo in the left inferior occipital gyrus (IOG), decreased fALFF and increased FC in the left inferior parietal lobule (IPL), and reduced cortical thickness in the right inferior temporal gyrus (ITG). Furthermore, functional and structural alterations in the SCD and aMCI groups were closely related to episodic memory (EM), executive function (EF), and information processing speed (IPS). The combination of multiple indicators of DAN had a high accuracy in differentiating clinical stages.

**Conclusions:**

Our current study demonstrated functional and structural alterations of DAN in SCD and aMCI, especially in the MTG, IPL, and SPL. Furthermore, cognitive performance was closely related to these significant alterations. Our study further suggested that the combined multiple indicators of DAN could be acted as the latent neuroimaging markers of preclinical and early‐stage AD for their high diagnostic value.

## Graphical abstract

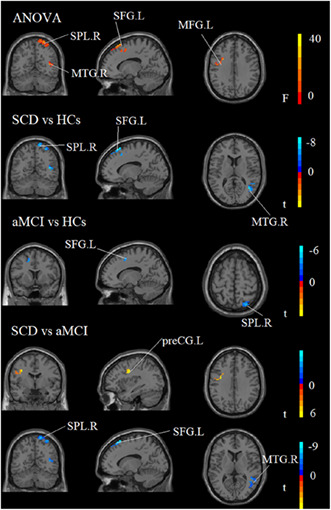

## 1 INTRODUCTION

Subjective cognitive decline (SCD) is known as the preclinical stage of Alzheimer's disease (AD).^1^ SCD is used to represent the condition of the elderly who are considered to show subjectively declined in the absence of objective cognitive impairment.^2^ Amnestic mild cognitive impairment (aMCI) is recognized as the early stage of AD.^3^ AMCI is a syndrome with cognitive degeneration, especially impaired memory.^4^ They are both high‐risk stages for conversion to AD.^5^ With the lack of effective treatment for AD, identifying sensitive diagnostic markers in the preclinical and early‐stage AD is crucial.^6^

Attention is a choice activity of consciousness, representing the initial link of cognitive processing.^7^ There has been a lot of evidence that early dementia patients have the clinical manifestations of attention deficit.^8^, ^9^, ^10^ As the most common neurodegenerative disease, the early clinical manifestation of AD is usually memory impairment. However, attention, as the initial stage of cognition, is often not easily perceived, which is also the starting point of this study. The resting‐state functional magnetic resonance imaging (rs‐fMRI) is an indispensable auxiliary diagnostic method to detect functional neuroimaging changes,^11^ especially in the brain networks.^12^ The dorsal attention network (DAN) has been associated with working and episodic memory and is mainly responsible for the “top‐down” attention process.^13^ Moreover, the latest research shows that DAN is highly predictive on cognitive ability relative to other networks.^14^

The fractional amplitudes of low‐frequency fluctuation (fALFF), regional homogeneity (ReHo), and functional connectivity (FC) are the three indicators measured by the rs‐fMRI.^15^ They, respectively, indicate the spontaneous activity of neurons,^16^ the consistency of neuronal activity,^17^ and neurophysiological activities in two or more spatially corresponding regions.^18^ Furthermore, structural magnetic resonance imaging (MRI) is also sensitive to examine structural brain alterations, including gray matter volume (GMV) and cortical thickness.^19^ In this article, we chose the combination of functional and structural measurements, a more comprehensive approach, for a better understanding of the neuroimages of DAN in SCD and aMCI.

Previous research mainly focused on the FC interactions between DAN and other networks in the spectrum of AD.^20^, ^21^ The explorations of DAN were simple with single functional modality and limited structure.^22^ Furthermore, previous studies lacked the changes of these indicators within DAN for the diagnosis and differentiation of SCD and aMCI. Taken over, these problems motivated us to extend previous research. We applied more functional indicators such as fALFF and ReHo and the structural indicators of GMV and cortical thickness. Additionally, we further explored the cognition associated with these alterations. We also employed the multimodal alterations of DAN to assess their diagnostic value in preclinical and early‐stage AD.

## 2 METHODS

### 2.1 Subjects

All subjects were from Nanjing Brain Hospital‐Alzheimer's Disease Spectrum Neuroimaging Project Version 2 (NBH‐ADsnp‐2), which has been approved by the responsible Human Participant Ethics Committee of the Affiliated Brain Hospital of Nanjing Medical University (2018‐KY010‐01, 2020‐KY010‐42).^23^, ^24^ All subjects in NBH‐ADsnp‐2, who were all Han Chinese and right‐handed, were recruited initially from hospitals and local communities by advertising and by means of broadcasting in the Appendix S1. All subjects participated voluntarily and obtained written informed consent. A total of 132 individuals from the baseline period of the NBH‐ADnsp‐2 database were initially enrolled. Among them, three aMCI participants, two SCD participants, and two HC participants were excluded due to excessive head motion (cumulative translation or rotation of >3.0 mm or 3.0°); 125 individuals were ultimately summarized in this study, including 40 aMCI, 43 SCD, and 42 healthy controls (HCs).

Inclusion criteria of SCD subjects were identified meeting the published SCD research criteria proposed by the Subjective Cognitive Decline Initiative (SCD‐I),^1^ and the detailed inclusion criteria have been described as follows: (a) self‐reported persistent memory decline, which was confirmed by an informant; (b) Subjective Cognitive Decline Questionnaire (SCD‐Q) score > 5^25^, ^26^, ^27^; (c) performance within the normal range on MMSE and MoCA (adjusted for age and education); (d) Clinical Dementia Rating (CDR) = 0; and (e) subjects aged between 50 and 80 years old.

Inclusion criteria of aMCI subjects were identified meeting the diagnostic criteria defined by Petersen et al.^28^ as well as the revised consensus standards presented by Winblad et al.,^29^ and the detailed inclusion criteria have been described as follows: (a) memory complaint preferably corroborated by an informant or the subject for more than 3 months; (b) objective memory impairment adjusted for age and educational level; (c) normal general cognitive function of MMSE score equal or above 24; (d) no or minimal impairment in daily living activities; (e) CDR = 0.5; (f) subjects aged between 50 and 80 years old; and (g) absence of dementia symptoms that were not sufficient to meet the criteria of the National Institute of Neurological and Communicative Disorders and Stroke or the AD and Related Disorders Association criteria for AD.

The detailed exclusion criteria for all subjects had been described in the Appendix S2.

### 2.2 Demographics and neuropsychological assessments

We collected demographic information (age, gender, and education levels) of all subjects. All subjects approved neuropsychological assessments by professional neuropsychologists. General cognitive function was assessed by Mini‐Mental State Examination (MMSE), Montreal Cognitive Assessment (MoCA), and Mattis Dementia Rating Scale‐2 (MDRS‐2). At the same time, the evaluation of four cognitive domains, including episodic memory (EM), executive function (EF), information processing speed (IPS), and visuospatial function (VF), was conducted based on neuropsychological assessments in Table 1. Division and assessment details were listed in the Appendix S3.

**TABLE 1:** Demographics and neuropsychological characteristics of HCs, SCD, and aMCI three groups.

	HCs (*n* = 42)	SCD (*n* = 43)	aMCI (*n* = 40)	*p*
Age (years)	63.82 (7.35)	63.63 (7.10)	64.65 (7.99)	0.673^a^
Gender (M/F)	16/26	10/33	18/22	0.104^b^
Education level (years)	12.36 (2.41)	11.85 (3.26)	11.89 (3.08)	0.679^a^
MMSE	29.00 (28.00, 30.00)	28.00 (27.00, 29.00)	27.50 (26.00, 29.00)	0.055^c^
MoCA	26.00 (25.00, 28.00)	25.00 (24.00, 27.00)	24.00 (22.00, 25.00)^d^ ^,*,^ ^e^ ^,**,^ ^f^ ^,***^	0.000^c^
MDRS‐2	142.00 (140.75, 144.00)	141.00 (139.00, 143.00)	138.00 (135.25, 141.00)^d^ ^,*,^ ^e^ ^,**,^ ^f^ ^,***^	0.001^c^
Composite *Z* scores of each cognitive domain				
Episodic memory	0.28 (0.59)	0.21 (0.62)	−0.52 (0.55)^e^ ^,***,^ ^f^ ^,***^	0.000^a^
Executive function	0.16 (0.53)	0.27 (0.52)	−0.46 (0.56)^e^ ^,***,^ ^f^ ^,***^	0.000^a^
Information processing speed	0.12 (0.75)	0.17 (0.70)	−0.31 (0.71)^e^ ^,**,^ ^f^ ^,*^	0.006^a^
Visuospatial function	0.30 (−0.18, 0.64)	0.15 (−0.37, 0.64)	0.01 (−0.50, 0.64)	0.141^c^

*Note*: Numbers are given as means (standard deviation, SD) or median (interquartile range, IQR).

Abbreviations: aMCI, amnestic mild cognitive impairment; HCs, healthy controls; MDRS‐2, Mattis Dementia Rating Scale‐2; MMSE, Mini‐Mental State Examination; MoCA, Montreal Cognitive Assessment test; SCD, subjective cognitive decline.

^a^ One‐way ANOVA.

^b^ Chi‐square test.

^c^ Kruskal‐Wallis *H*‐test.

^d^ Post hoc analyses showed a significant group difference between HCs and SCD.

^e^ Post hoc analyses showed a significant group difference between SCD and aMCI.

^f^ Post hoc analyses showed a significant group difference between HCs and aMCI.

*p < 0.05, **p < 0.01, ***p < 0.001.

### 2.3 Image acquisition

All MRI scans were performed on a 3.0T scanner. The detailed image acquisition parameters, including rs‐fMRI images and structural MRI images, were provided in the Appendix S4.

### 2.4 Rs‐fMRI data preprocessing

All rs‐fMRI data were preprocessed in the matlab2013b (http://www.mathworks.com/products/matlab/) and Data Processing Assistant for Resting‐State FMRI (DPARSF, http://restfmri.net/forum/DPARSF).^30^ The first 10 time points were removed before slice time and head motion correction. Then, we detached subjects with excessive head motion (cumulative translation or rotation >3.0 mm or 3.0°). Subsequently, structural images were segmented into gray matter (GM), white matter (WM), and cerebrospinal fluid (CSF) partitions. We used the Diffeomorphic Anatomical Registration Through Exponentiated Lie (DARTEL) algorithm to normalize.^31^ After normalization, they were regressed with the Friston 24‐parameter model and resampled 3 × 3 × 3 mm^3^ into Montreal Neurological Institute (MNI). Finally, the fMRI data were spatially smoothed and temporal band‐pass filtered.^32^

### 2.5 Structural MRI data preprocessing

The structural T1 images were preprocessed in the Data Processing Analysis Brain Imaging (DPABI).^30^ The structural images were segmented into GM, WM, and CSF. The resolution of the resulting GM images was 1.5 × 1.5 × 1.5 mm^3^. Then, spatially smoothed by a Gaussian kernel of 6 mm^3^ full‐width at half maximum (FWHM) to reduce spatial noise. The FreeSurfer software also analyzed the cortical thickness with T1 images. The standard processing procedures included were in the Appendix S5.

### 2.6 Definition of DAN mask

According to the previous meta‐analysis, the brain areas of DAN were summarized.^15^ Then, we used the WFU_PickAtlas toolbox (www.ansir.wfubmc.edu) in the matlab2013b to make the DAN mask. The DAN mask included bilateral precentral gyrus (PreCG), superior frontal gyrus (SFG), middle frontal gyrus (MFG), superior parietal lobule (SPL), intraparietal sulcus (IPS), inferior occipital gyrus (IOG), inferior temporal gyrus (ITG), and MTG.^33^, ^34^, ^35^, ^36^, ^37^, ^38^, ^39^

### 2.7 FALFF, ReHo, and FC measurement

The calculation of the fALFF value was performed in the DPABI software. The fALFF data were temporally band‐pass filtered (from 0.01 to 0.10 Hz) to reduce the low‐frequency drift and high‐frequency respiratory and cardiac noise. For each given voxel, the time series were converted to the frequency domain, and the power spectrum was obtained. The fALFF results were transformed using Fisher's z transformation fractional amplitude of low‐frequency fluctuation (zfALFF) and used for the following analysis.^40^

The ReHo index was computed in a voxel‐wise manner using Kendall's correlation coefficient of a given voxel and its adjacent voxel (26 voxels) from the unsmoothed time series. Each ReHo map was also transformed using Fisher's z transformation regional homogeneity (zReHo). These zReHo maps were spatially smoothed with a 6‐mm Gaussian kernel FWHM to reduce the effect of noise. We earned smoothed zReHo (szReHo) and used it for subsequent analysis.^41^

Seed‐based FC analysis was carried out to explore the alternations of DAN. According to a previous study, the right IPS was a vital region in the DAN. We made a 6‐mm spherical region of interest (ROI) centered in the right IPS (MNI space: 32, −56, 54).^42^, ^43^ The voxel‐wise Pearson correlation analysis was extracted between the average time courses of right IPS and the whole brain within the GM mask. Then, the Fisher's z conversion was executed for subsequent analysis.^44^

### 2.8 Statistical analysis

Statistical Package for Social Sciences (SPSS) software (version 26) was used to perform statistical analysis. In demographic and neuropsychological characteristics, continuous variables were expressed as mean ± standard deviation (SD), and discontinuous variables were expressed as median ± interquartile range (IQR). Kolmogorov–Smirnov test (*n* > 50) or Shapiro–Wilk test (*n* ≤ 50) was used to evaluate the normality hypothesis of the data. The one‐way analysis of variance (ANOVA) was used to analyze the differences of continuous variables with normal distribution among the three groups, and Kruskal–Wallis *H*‐test was used to analyze continuous variables without normal distribution. The post hoc test was obtained by two‐sample *t*‐test Bonferroni correction or Mann‐Whitney *U*‐test (*p* < 0.05). The chi‐square test was used for the difference in gender among the three groups.

Using DPABI software, the ANOVA test within the DAN mask was to compare the differences in fALFF, ReHo, and GMV among these three groups, the age, gender, and years of education as covariates. The nonparametric permutation test (1000 permutations) was conducted, and the significance level was set to *p* < 0.05. Post hoc comparisons using the two‐sample *t*‐test with the mask resulted from ANOVA analyses were performed after controlling the effects of age, gender, and years of education. The significance level was set with TFCE‐FWE corrected at *p* < 0.05 in fALFF and ReHo, and TFCE‐FDR corrected at *p* < 0.05 in GMV.

A one‐way ANOVA analysis within GM mask was conducted using DPABI to compare the differences in FC across three groups, including aMCI, SCD, and HCs, with age, gender, and years of education as covariates. The nonparametric permutation test (1000 permutations) was conducted, and the significance level was set to *p* < 0.05. The two‐sample *t*‐test was used for post hoc comparisons with the mask resulting from ANOVA analyses after controlling the effects of age, gender, and years of education. The TFCE‐FDR correction was applied with a threshold of *p* < 0.05 in FC.

The Pearson correlation analysis was conducted to explore the relationship between functional and structural alterations and cognitive performance after controlling the effects of age, gender, and years of education in the SPSS software. Moreover, we evaluated the sensitivity and specificity of all meaningful biomarkers in combination to predict their value in SCD and aMCI with ROC curves.

## 3 RESULTS

### 3.1 Demographic and neuropsychological characteristics

Table 1 summarizes the demographic and neuropsychological characteristics of 125 participants in three groups, including 42 HCs (mean age 63.82 ± 7.35), 43 SCD (mean age 63.63 ± 7.10), and 40 aMCI (mean age 64.65 ± 7.99). As is expected, the results showed significant differences in cognitive performance. Compared with HCs and SCD, the aMCI group showed lower MMSE, MoCA, and MDRS‐2 (Bonferroni corrected for post hoc, all *p* < 0.05). At the same time, the aMCI group also showed significantly lower EM, EF, and IPS (Bonferroni corrected for post hoc, all *p* < 0.05) than HCs and SCD.

### 3.2 FALFF, ReHo, and FC alterations of DAN in SCD and aMCI

**FIGURE 1:**
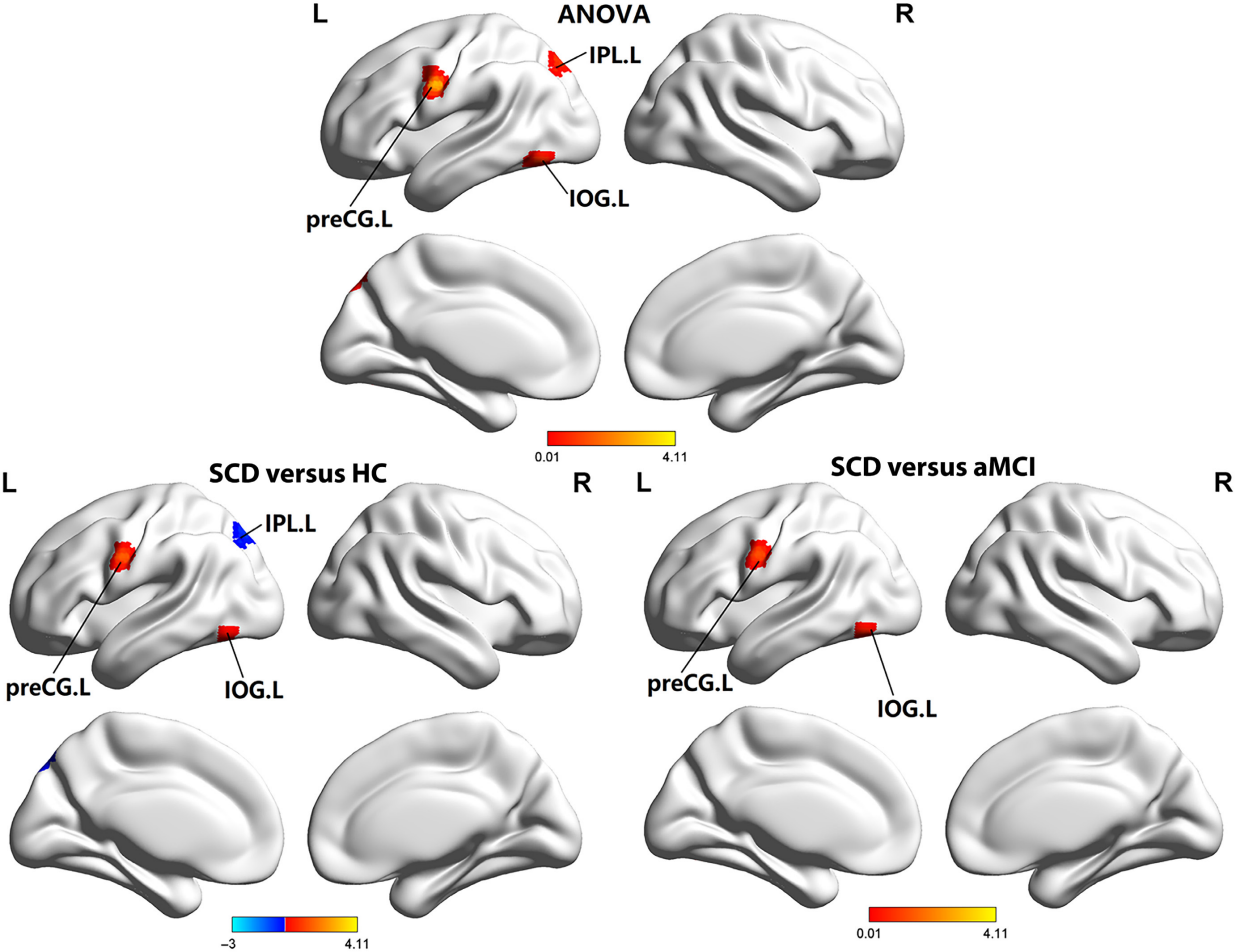
Brain regions showed significant differences in fALFF of the DAN across three groups. aMCI, amnestic mild cognitive impairment; HCs, healthy controls; IOG, inferior occipital gyrus; IPL, inferior parietal lobule; L, left; preCG, precentral gyrus; R, right; SCD, subjective cognitive decline.

The fALFF ANOVA analysis had significant differences in some brain regions among HCs, SCD, and aMCI groups, including the left IOG, left inferior parietal lobule (IPL), and left preCG. Compared to HCs, the SCD group displayed increased alterations in left preCG and left IOG and decreased changes in the left IPL, respectively. In comparison with aMCI, increased fALFF values in the left IOG and left preCG were significant in the SCD group (see Table 2 and Figure 1).

**TABLE 2:** FALFF values across HCs, SCD, and aMCI groups.

Region (aal)	Peak MNI coordinate			*F*/*t*	Cluster number
	*x*	*y*	*z*		
ANOVA					
L inferior occipital gyrus	−51	−69	−18	8.4755	31
L inferior parietal lobule	−30	−75	48	6.248	30
L precentral gyrus	−54	−3	30	11.0853	20
SCD > HCs					
L precentral gyrus	−54	−3	30	4.1092	15
L inferior occipital gyrus	−48	−69	−12	2.6455	9
SCD < HCs					
L inferior parietal lobule	−30	−75	48	−3.0031	26
SCD > aMCI					
L inferior occipital gyrus	−51	−69	−18	3.8032	21
L precentral gyrus	−54	−3	30	3.5988	15

*Note*: Brain regions showed significant differences in fALFF of the DAN across three groups (TFCE‐FWE corrected, cluster size >15 voxels, *p* < 0.05) and results of post hoc analysis in voxel‐wise analysis (Bonferroni corrected, cluster size >5 voxels, *p* < 0.05).

Abbreviations: aMCI, amnestic mild cognitive impairment; HCs, healthy controls; L, left; R, right; SCD, subjective cognitive decline.

**FIGURE 2:**
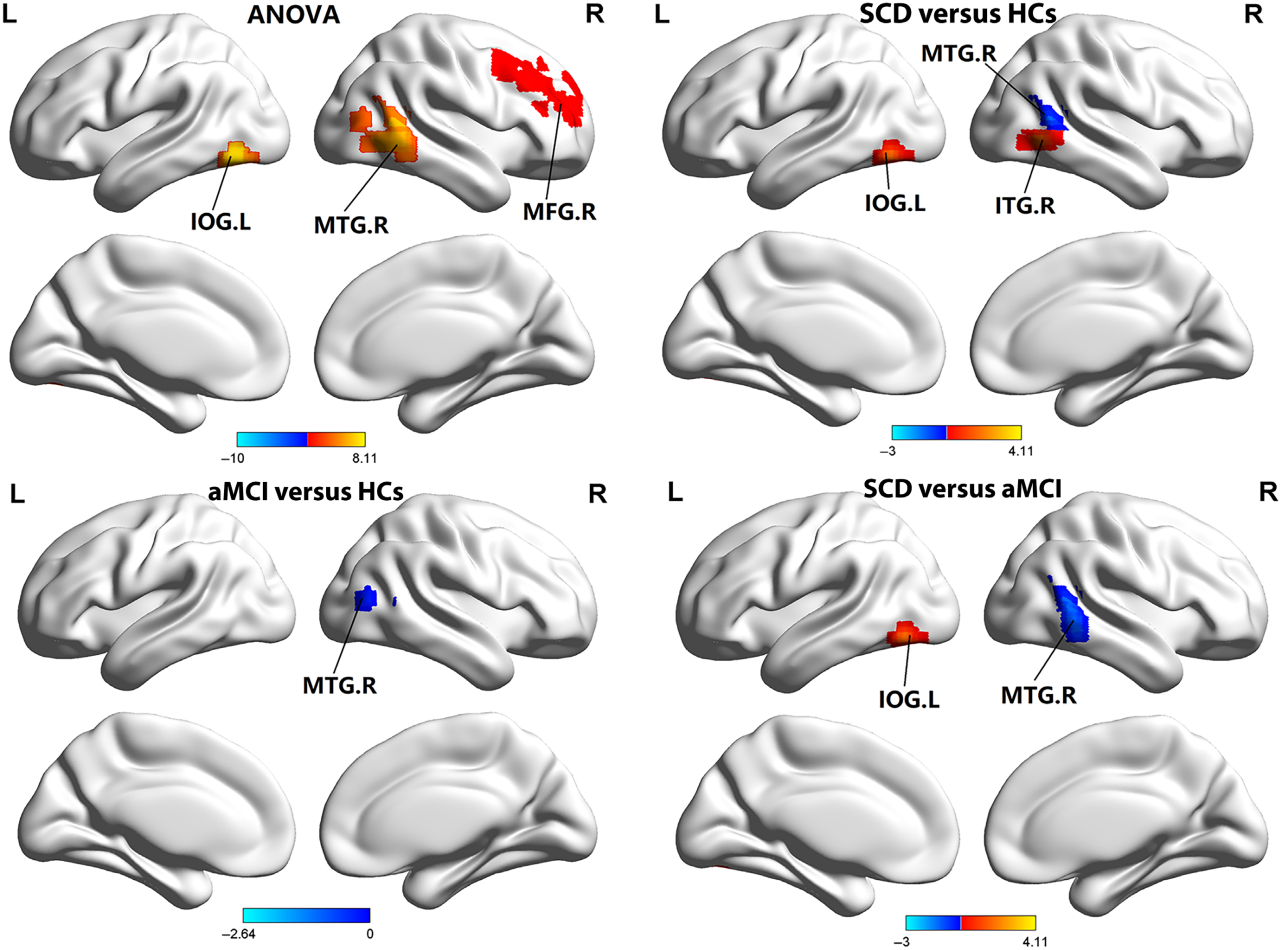
Brain regions showed significant differences in ReHo of the DAN across three groups. aMCI, amnestic mild cognitive impairment; HCs, healthy controls; IOG, inferior occipital gyrus; L, left; MFG, middle frontal gyrus; MTG, middle temporal gyrus; R, right; SCD, subjective cognitive decline.

In the ReHo ANOVA analysis, significant results were mainly in the right MFG, right MTG, and left IOG among three groups. Compared to HCs, the SCD and aMCI groups showed decreased ReHo values in the right MTG. Incredibly, there was an increase in the SCD group in the left IOG, and right ITG compared to HCs. Besides, the aMCI group exhibited significantly increased ReHo values in the right MTG and decreased in the left IOG in comparison with SCD (see Table 3 and Figure 2).

**TABLE 3:** ReHo values across HCs, SCD, and aMCI groups.

Region (aal)	Peak MNI coordinate			*F*/*t*	Cluster number
	*x*	*y*	*z*		
ANOVA					
R middle frontal gyrus	54	24	39	0.12798	227
R middle temporal gyrus	60	−66	15	7.7837	145
L inferior occipital gyrus	−54	−69	−12	8.1143	49
SCD > HCs					
L inferior occipital gyrus	−48	−69	−12	3.253	43
R inferior temporal gyrus	51	−63	−3	3.3779	33
SCD < HCs					
R middle temporal gyrus	60	−66	15	−3.436	71
aMCI < HCs					
R middle temporal gyrus	60	−63	15	−2.6433	15
SCD > aMCI					
L inferior occipital gyrus	−54	−69	−12	4.1794	48
SCD < aMCI					
R middle temporal gyrus	57	−51	12	−3.2725	54

*Note*: Brain regions showed significant differences in ReHo of the DAN across three groups (TFCE‐FWE corrected, cluster size >40 voxels, *p* < 0.05) and results of post hoc analysis in voxel‐wise analysis (Bonferroni corrected, cluster size >10 voxels, *p* < 0.05).

Abbreviations: aMCI, amnestic mild cognitive impairment; HCs, healthy controls; L, left; R, right; SCD, subjective cognitive decline.

**FIGURE 3:**
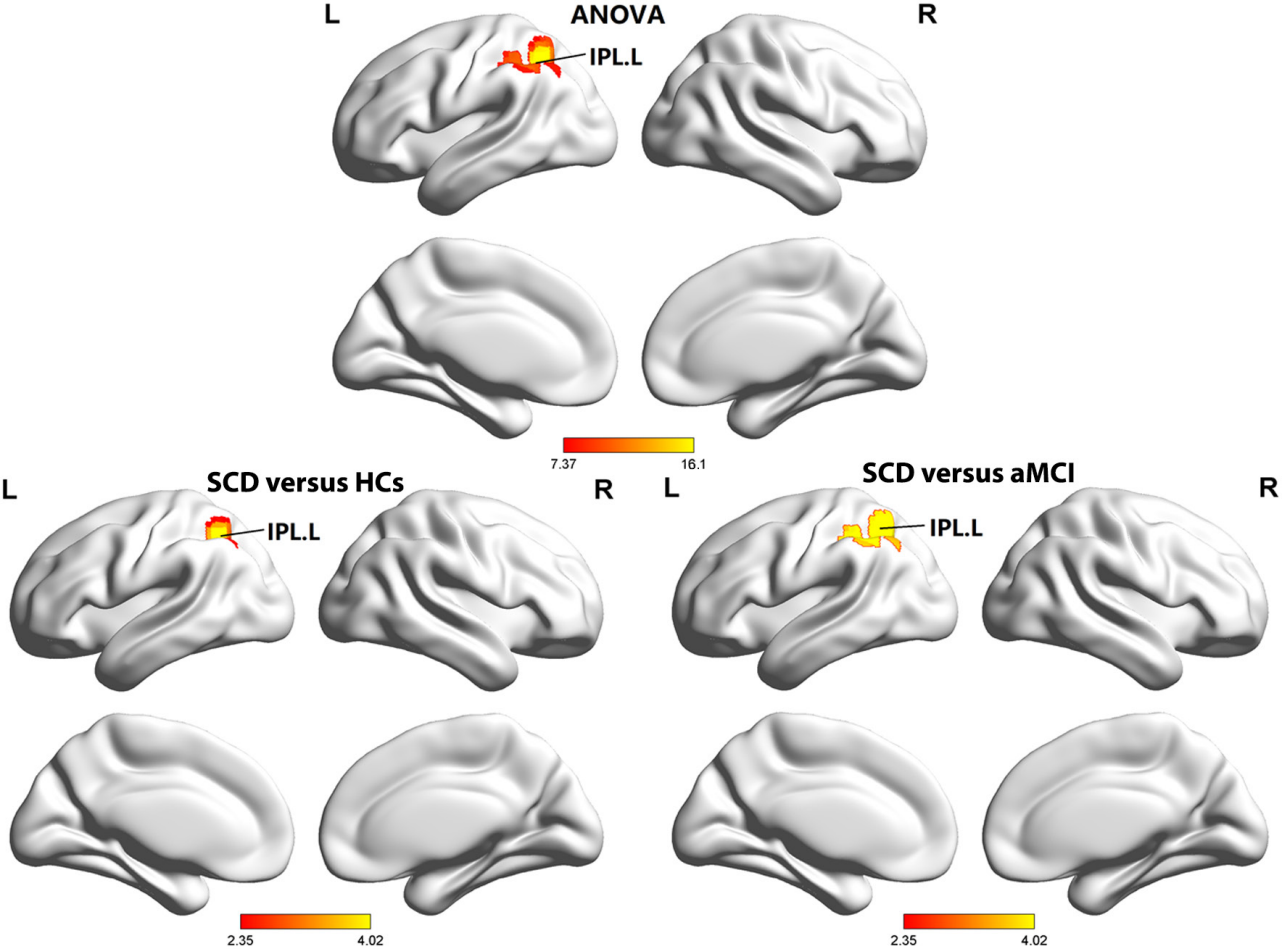
Brain regions showed significant differences in FC of the DAN across three groups. aMCI, amnestic mild cognitive impairment; HCs, healthy controls; IPL, inferior parietal lobule; L, left; R, right; SCD, subjective cognitive decline.

The ANOVA analysis showed significant alterations across these three groups concerning the FC aspect, especially in the left IPL. Compared to HCs, increased FC values in the SCD group were significant in the left IPL. The aMCI group showed significantly decreased FC values in the left IPL (see Table 4 and Figure 3).

**TABLE 4:** FC values across HCs, SCD, and aMCI groups.

Region (aal)	Peak MNI coordinate			*F*/*t*	Cluster number
	*x*	*y*	*z*		
ANOVA					
L inferior parietal lobule	−30	−57	48	16.0974	76
L inferior parietal lobule	−42	−51	33	12.4807	56
SCD > HCs					
L inferior parietal lobule	−30	−57	48	4.0238	39
SCD > aMCI					
L inferior parietal lobule	−30	−57	51	5.0537	76
L inferior parietal lobule	−42	−45	48	4.3787	56

*Note*: Brain regions showed significant differences in FC of the DAN across three groups (TFCE‐FDR corrected, cluster size >50 voxels, *p* < 0.05) and results of post hoc analysis in voxel‐wise analysis (Bonferroni corrected, cluster size >30 voxels, *p* < 0.05).

Abbreviations: aMCI, amnestic mild cognitive impairment; HCs, healthy controls; L, left; R, right; SCD, subjective cognitive decline.

### 3.3 GMV and cortical thickness alterations of DAN in SCD and aMCI

**FIGURE 4:**
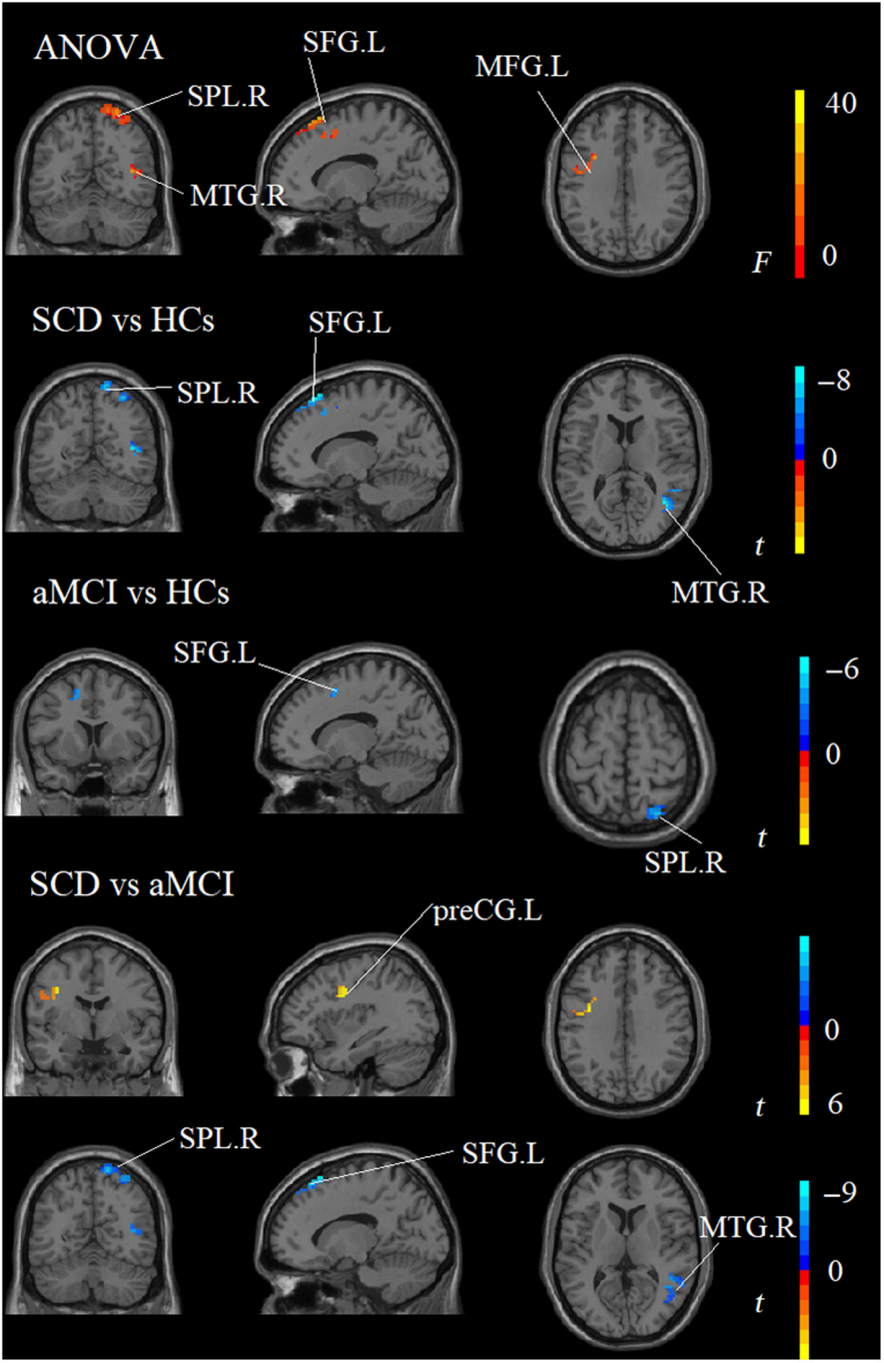
Brain regions showed significant differences in GMV of the DAN across three groups. aMCI, amnestic mild cognitive impairment; HCs, healthy controls; L, left; MFG, middle frontal gyrus; MTG, middle temporal gyrus; R, right; SCD, subjective cognitive decline; SFG, superior frontal gyrus; SPL, superior parietal lobule.

The ANOVA analysis showed significant changes in GMV of DAN across three groups, including the left MFG, right SPL, right MTG, and left SFG. Compared to HCs, the SCD and aMCI groups showed decreased GMV values in the left SFG and right SPL. The aMCI group exhibited an increase in the right MTG, left SFG, and right SPL and a decrease in the left preCG compared to SCD (see Table 5 and Figure 4).

**TABLE 5:** GMV values across HCs, SCD, and aMCI groups.

Region (aal)	Peak MNI coordinate			*F*/*t*	Cluster number
	*x*	*y*	*z*		
ANOVA					
L middle frontal gyrus	−30	9	33	26.1047	143
R superior parietal lobule	24	−60	72	30.7415	112
R middle temporal gyrus	39	−60	12	27.6392	88
L superior frontal gyrus	−15	24	66	38.0666	85
SCD < HCs					
R middle temporal gyrus	39	−60	12	−7.7543	40
L superior frontal gyrus	−15	24	66	−7.2529	35
R superior parietal lobule	15	−60	75	−5.7876	24
aMCI < HCs					
L superior frontal gyrus	−15	9	51	−4.8495	43
R superior parietal lobule	24	−69	63	−5.1014	30
SCD < aMCI					
R middle temporal gyrus	39	−54	9	−4.8384	72
R superior parietal lobule	15	−60	75	−5.9125	57
L superior frontal gyrus	−15	24	66	−8.7607	43
SCD > aMCI					
L precentral gyrus	−36	−3	33	5.8708	44

*Note*: Brain regions showed significant differences in GMV of the DAN across three groups (TFCE‐FDR corrected, cluster size >80 voxels, *p* < 0.05) and results of post hoc analysis in voxel‐wise analysis (Bonferroni corrected, cluster size >20 voxels, *p* < 0.05).

Abbreviations: aMCI, amnestic mild cognitive impairment; HCs, healthy controls; L, left; R, right; SCD, subjective cognitive decline.

Regarding cortical thickness, the ANOVA analysis showed significant differences across these three groups in the right ITG. Compared to HCs, decreased signal in the right ITG was particularly in the SCD group (Bonferroni corrected for post hoc, *p* < 0.05) (see Table 6).

**TABLE 6:** Cortical thickness across HCs, SCD, and aMCI groups.

Cortical thickness	HCs	SCD	aMCI	*F*	*p*
L Superior parietal lobule	2.198 (0.113)	2.195 (0.128)	2.192 (0.141)	0.024	0.976
R Superior parietal lobule	2.174 (0.103)	2.165 (0.136)	2.172 (0.140)	0.063	0.939
L Inferior parietal lobule	2.466 (0.119)	2.434 (0.105)	2.449 (0.144)	0.728	0.485
R Inferior parietal lobule	2.465 (0.117)	2.444 (0.110)	2.449 (0.143)	0.329	0.721
L Middle temporal gyrus	2.749 (0.103)	2.741 (0.129)	2.778 (0.143)	0.952	0.389
R Middle temporal gyrus	2.815 (0.120)	2.813 (0.129)	2.822 (0.129)	0.058	0.944
L Inferior temporal gyrus	2.889 (0.096)	2.835 (0.159)	2.854 (0.149)	1.670	0.192
R Inferior temporal gyrus	2.880 (0.129)	2.803 (0.126)	2.866 (0.144)	4.040	0.020^a^
Precentral	2.529 (0.140)	2.556 (0.173)	2.505 (0.206)	0.895	0.411
R Precentral	2.508 (0.141)	2.527 (0.172)	2.467 (0.193)	1.311	0.273

*Note*: Numbers are given as means (standard deviation, SD) unless stated otherwise. Brain regions showed significant differences in the cortical thickness of the DAN across three groups (all *p* < 0.05) and results of post hoc analysis (Bonferroni corrected, *p* < 0.05).

Abbreviations: aMCI, amnestic mild cognitive impairment; HCs, healthy controls; L, left; R, right; SCD, subjective cognitive decline.

^a^ Post hoc analysis showed a significant difference between SCD and HCs.

### 3.4 Pearson correlation analysis

**FIGURE 5:**
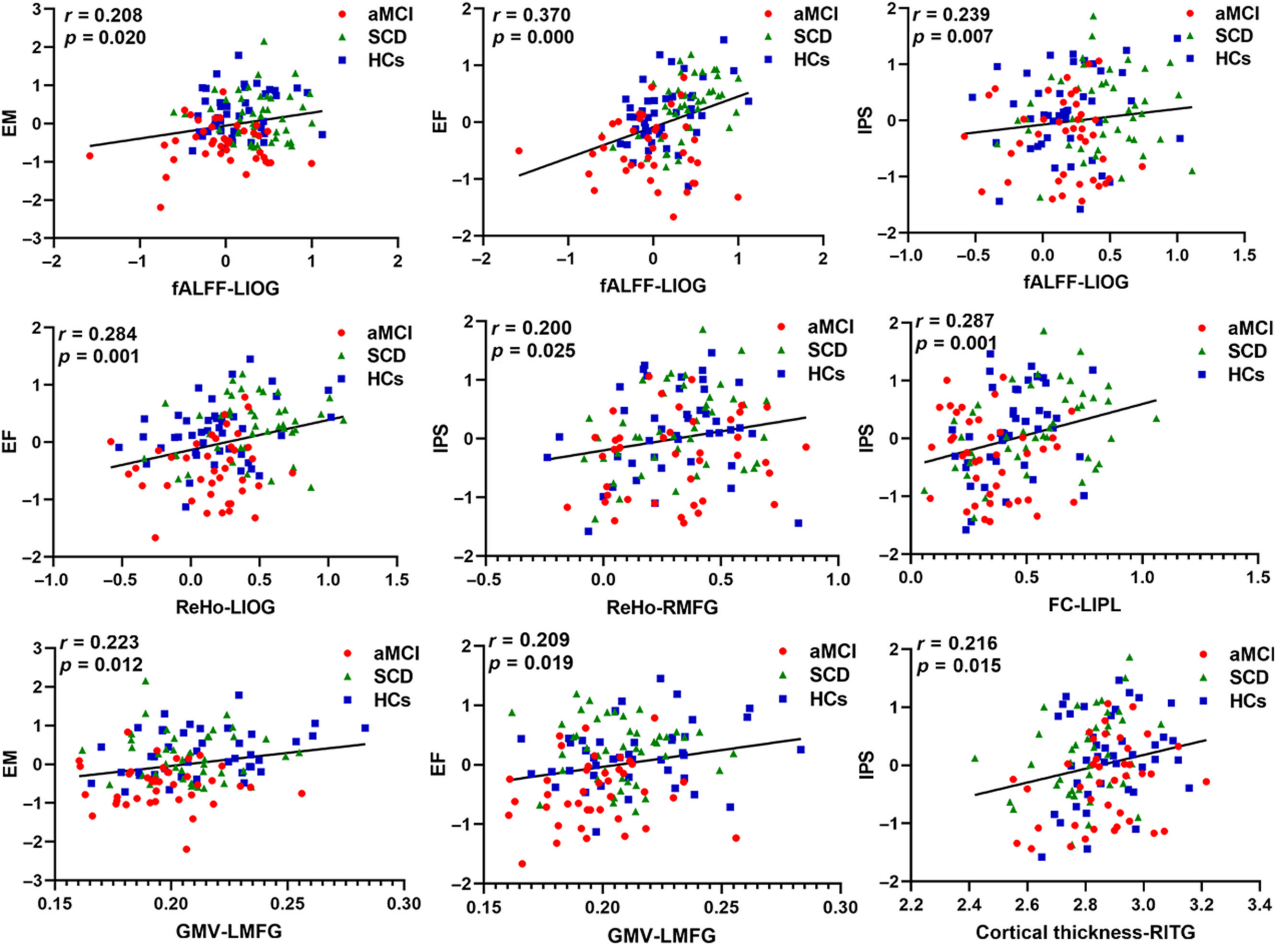
Pearson correction analysis between cognitive performance and functional and structural alterations of DAN in HCs, SCD, and aMCI groups. aMCI, amnestic mild cognitive impairment; fALFF, fractional amplitude of low‐frequency fluctuation; FC, functional connectivity; GMV, gray matter volume; HCs, healthy controls; IOG, inferior occipital gyrus; IPL, inferior parietal lobule; ITG, inferior temporal gyrus; L, left; MFG, middle frontal gyrus; R, right; ReHo, regional homogeneity; SCD, subjective cognitive decline.

Pearson correlation analysis was conducted between functional and structural alterations in DAN and neuropsychological scales (Figure 5, all *p* < 0.05). This study mainly focused on analyzing HCs, SCD, and aMCI groups. The analysis showed that the fALFF value in the left IOG was positively correlated with EM, EF, and IPS scores (*r* = 0.208, *p* = 0.020; *r* = 0.370, *p* = 0.000; *r* = 0.239, *p* = 0.007). The ReHo value in the left IOG and right MFG were also positively correlated with the EF score (*r* = 0.284, *p* = 0.001) and IPS score (*r* = 0.200, *p* = 0.025). Moreover, the altered FC in the left IPL was positively correlated with IPS score (*r* = 0.287, *p* = 0.001). The GMV value in the left MFG was positively correlated with EM and EF (*r* = 0.223, *p* = 0.012; *r* = 0.209, *p* = 0.019). The cortical thickness value in the right ITG was positively correlated with IPS (*r* = 0.216, *p* = 0.015).

### 3.5 ROC analysis

**FIGURE 6:**
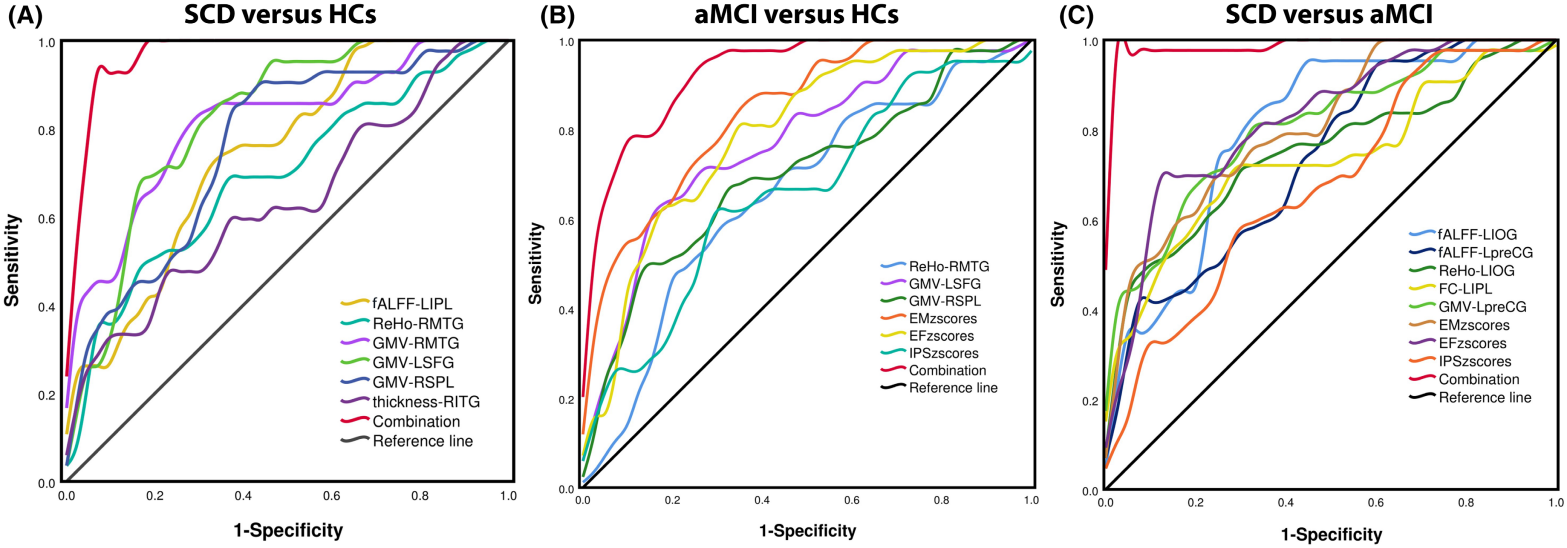
Classification of individuals across three groups on ROC analysis. (A) ROC curve presenting the classification of SCD vs. HCs; (B) ROC curve presenting the classification of aMCI vs. HCs; (C) ROC curve presenting the classification of SCD vs. aMCI; aMCI, amnestic mild cognitive impairment; HCs, healthy controls; L, left; R, right; SCD, subjective cognitive decline.

In the groups of SCD and HCs, the area under the curve (AUC) values of fALFF in the left IPL and ReHo in the right MTG were 0.744 and 0.692. The GMV in the right MTG, left SFG, and right SPL had the AUC values of 0.815, 0.823, and 0.761. The AUC values of cortical thickness in the right ITG were 0.636 (all *p* < 0.05, Figure 6A).

In the groups of aMCI and HCs, the AUC values of ReHo in the right MTG and GMV in the left SFG and right SPL were 0.658, 0.770 and 0.699. The EM, EF, and IPS had the AUC values of 0.840, 0.791, and 0.660 (all *p* < 0.05, Figure 6B).

In the groups of SCD and aMCI, the AUC values of fALFF in the left IOG and left preCG were 0.802 and 0.737. The AUC values of ReHo in the left IOG and altered FC in the left IPL were 0.751 and 0.763. The EM, EF, and IPS had the AUC values of 0.810, 0.824, and 0.677 (all *p* < 0.05, Figure 6C).

## 4 DISCUSSION

This study investigated functional and structural alterations of DAN in SCD and aMCI groups comprehensively. According to our findings, fALFF, ReHo, FC, GMV, and cortical thickness had significant changes. Furthermore, the combined multiple indicators of DAN can be acted as the latent neuroimaging markers of preclinical and early‐stage AD for their high diagnostic value.

### 4.1 Altered functional patterns of DAN in SCD and aMCI

As presented, fALFF alterations were mainly located in the left IOG, left IPL, and left preCG. As we all know, IOG is associated with cognitive performance, with a previous PET study showing a significant increase in metabolism in the occipital cortex in cognitive impairment.^45^ A previous study reported that the SCD group exhibited higher fALFF values than HCs, consistent with our study.^46^ The function of preCG is to start the purposeful movement by integrating the information through the sensory‐motor cortex.^47^ Increased fALFF values indicated enhanced neuronal connectivity in the left IOG and left preCG in the process of cognitive impairment.^48^ IPL has a close connection with episodic memory.^49^ Compared to HCs, the SCD group exhibited a significant decrease in fALFF values in the IPL, further confirming the continuous episodic memory decline from HCs to SCD.^50^

With regard to ReHo values, right MTG and left IOG were shown significant alterations. Previous studies had found that the MTG was crucial in many cognitive domains such as language processing and deductive reasoning.^51^ Compared to HCs, the SCD group showed lower ReHo values, which meant a greater deficit in cognitive performance.^50^ The ReHo values in the left IOG showed increased alterations, which was the same as the fALFF. The increased value indicated that the neuronal activity in the local brain area tended to increase.^52^ A study reported that neurodegeneration caused the accumulation of amyloid plaques to overexcite neurons.^53^ This research could suggest that the increased ReHo in the left IOG may play an essential role in early functional compensation.

As for FC, the most apparent change was mainly in the left IPL. IPL integrates information from different sensory modalities and plays an essential role in various higher cognitive functions.^54^ Compared to HCs and aMCI, the SCD group showed increased FC in the left IPL. IPL was the classic brain region of the DAN. The increased connectivity in left IPL was within the DAN, which meant the increased activity of the DAN itself to some extent.^55^

### 4.2 Altered structural patterns of DAN in SCD and aMCI

Regarding the GMV values, the reduction in the SCD and aMCI groups was mainly in the left MFG, right SPL, right MTG, and left SFG. A study reported that the GMV of MFG was significantly correlated with memory performance.^56^ The decreased GMV in MFG can affect the processing of word understanding in the elderly.^57^ SPL has also been related to processing movement in space, and the GMV in SPL declined with the progression of AD.^58^ Surprisingly, a study reported that those participants who spent more time‐performing sports activity per week showed higher GMV in SPL.^59^ In this way, we can prevent AD progression through sports activity. The research informed that the GMV showed a noticeable decline in the MTG.^56^ The subregions of MTG are related to human episodic memory.^51^ A study also indicated that primary foci of atrophy were identified in the MTG in AD patients.^60^ Thus, the atrophy of MTG could be used as a predictive factor for AD progression. The SFG has been associated with fluency tasks. Especially in aMCI, the SFG predicted damaged memory performance in both category and design fluency.^61^

The cortical thickness of right ITG between SCD and HCs showed a significant decrease. The ITG plays an essential role in verbal fluency. Individuals with cognitive impairment had significantly fewer synapses in the ITG than individuals with no cognitive impairment. These results demonstrated that the ITG was affected during the early stage of the disease and may underlie some of the early AD‐related clinical dysfunctions.

### 4.3 Cognitive performance and alterations of DAN in SCD and aMCI

In terms of cognitive performance, Pearson correlation analysis showed that the decreased fALFF values in the left IOG were accompanied by decreased EM, EF, and IPS. At the same time, decreased ReHo in the left IOG also had a relationship with decreased EF. The occipital lobe lesions are often associated with visuospatial‐structural dysfunction, usually occurring in the early stages of AD. The study showed that the functional value in the occipital lobe was reduced, related to memory loss.^62^ The positive relation between the cognitive performance and FC in the left IPL was clear for its role in cognitive processes.^63^ The decreased GMV in preCG in SCD and aMCI was related to cognitive dysfunction. Voxel‐level GMV analysis revealed strong relationships between volumes of the preCG and olfactory identification impairments, which frequently occurred in the AD disease.^64^ The results demonstrated the involvement of DAN in cognitive function, consistent with previous studies.^65^, ^66^ Moreover, it further confirmed the functional and structural alterations in DAN had a significant connection with cognitive function.

Through the ROC analysis, the most important finding in this study was that the neuroimages of DAN across the three groups had a very high accuracy. The AUC values of GMV in the left SFG and right SPL were up to 0.815 (sensitivity = 85.7%, specificity = 67.4%) and 0.823 (sensitivity = 90.5%, specificity = 58.1%) in SCD and HCs. The AUC value of EM in aMCI and HCs was 0.84 (sensitivity = 83.3%, specificity = 67.5%) in aMCI and HCs. The AUC values of left IOG, EM, and EF were 0.802 (sensitivity = 95.3%, specificity = 57.5%), 0.810 (sensitivity = 69.8%, specificity = 77.5%), and 0.824 (sensitivity = 69.8%, specificity = 90%) in SCD and aMCI. All results showed the advantages of the functional and structural alterations of DAN in studying preclinical and early‐stage AD and provided a new sight for the diagnosis and differentiation in the progression of AD.

### 4.4 Limitations

There were the following notable limitations in this study. First of all, we had a small sample size, and only 42 HCs, 43 SCD, and 40 aMCI were included in our study. At the same time, our NBH‐ADsnp‐2 database is being expanded, and more volunteers are being recruited. Therefore, we will further fill the gap of a small sample size. Secondly, this study was just a baseline study. A longitudinal study with further follow‐up of these data is required to evaluate the alterations of neuroimaging markers in AD progression comprehensively. Moreover, the gender distribution in these three groups was unbalanced, which may lead to bias in our results. Fortunately, we took age, gender, and education as covariates in the process of statistical analyses in order to reduce the impact to a certain extent. Lastly, the plasma biomarker of early‐stage AD spectrum plays an important role in clinical diagnosis. In the future, we will pay more attention to the plasma biomarker to expand the significance of research.

## 5 CONCLUSION

Our current study demonstrated obviously functional and structural alterations of DAN in SCD and aMCI. We found that the abnormalities of the functional alterations were especially located in the IPL, IOG, and MTG, whereas structural alterations were mainly in the SPL and ITG. Furthermore, cognitive performance was closely related to these significant alterations. Our study further suggested that the combined multiple indicators of DAN could be acted as the latent neuroimaging markers of preclinical and early‐stage AD for their high diagnostic value.

## AUTHOR CONTRIBUTIONS

HW, YS, XY, XL, and JC: designed the study. HW, YS, XY, CS, HG, ZY, WQ, QY, and XL: collected the data. HW, YS, and XY: analyzed the data and prepared the manuscript. XL and JC modified the article and approved the submission. HW, YS, and XY contributed equally (joint first authors).

## CONFLICT OF INTEREST

The authors declare that they have no competing financial interests.

## Supporting information


Appendix S1–S5

## Data Availability

The data that support the findings of this study are available from the corresponding author upon reasonable request.
