# Supplementary material for: Functional and structural alterations of dorsal attention network in preclinical and early‐stage Alzheimer's disease
Source: CNS Neurosci Ther. 2023 Mar 21;29(6):1512–24. doi: 10.1111/cns.14092 (PMC10173716; doi:10.1111/cns.14092)
Supplement: Supplementary file 1 — Appendix S1–S5 [file CNS-29-1512-s001.doc]

**Supplementary Material**

**S.1 NBH-ADsnp-2 Database**

Data used in this study were obtained from the Nanjing Brain Hospital-Alzheimer’s Disease Spectrum Neuroimaging Project Version 2 (NBH-ADsnp-2) database. NBH-ADsnp-2 is an upgrade of NBH-ADsnp and is derived from an Alzheimer’s Disease Spectrum Neuroimaging cooperative Project that was jointly built in September 2022 by Department of Radiology, Nanjing Drum Tower Hospital and Department of Radiology and Neurology, the Affiliated Brain Hospital of Nanjing Medical University. Prof. Jiu Chen, PhD, MD, from Nanjing Drum Tower Hospital, and Xingjian Lin, MD and Chaoyong Xiao, MD, from the Affiliated Brain Hospital of Nanjing Medical University, acts as the principal investigator of NBH-ADsnp-2. NBH-ADsnp-2 was initiated by Dr. Jiu Chen, Dr. Xingjian Lin, and Dr. Chaoyong Xiao and was named by Dr. Chen, Dr. Lin, Dr. Xiao's cooperative research group (discussed by Chen Xue, Guan-jie Hu, Wen-wen Xu, Wan Liu, Wen-zhang Qi, Si-yu Wang, Jia-ni Xu, Shan-shan Chen, Honglin Ge, Zheng Yan, Yu Song, Qianqian Yuan, Huimin Wu, Xuhong Liang, Xinyi Yang and finally verified by Jiu Chen, Xingjian Lin, and Chaoyong Xiao). NBH-ADsnp-2 is an observational and intervention study which includes cross-sectional and longitudinal follow-up components. The goal of NBH-ADsnp-2 is to identify early neuroimaging biomarkers of preclinical Alzheimer’s Disease (AD) spectrum {subjective cognitive decline (SCD), amnestic mild cognitive impairment (aMCI), amnestic mild cognitive impairment (naMCI), and AD}, to predict the disease progression of individuals within preclinical AD spectrum, and to provide imaging-based targets for individualized intervention in order to prevent the disease deterioration from preclinical stages to the eventually progressed AD. All subjects in NBH-ADsnp-2, who were all Han Chinese and right-handed, were recruited initially from hospitals and local communities by advertising and by means of broadcasting. This database used a standardized clinical evaluation protocol that included a medical history interview, neurologic examination, a battery of neurocognitive assessment, and resting-state MRI scan (T1, T2, 3D T1, DTI, and BOLD) for all participants (healthy controls, SCD, naMCI, aMCI, and AD). In addition, MRI data collected after 2 and 4 weeks of rTMS intervention in patients with SCD and MCI were added after the database upgrade. All subjects and their study partners completed the informed consent process, and the study protocols were reviewed and approved by the responsible Human Participants Ethics Committee of the Affiliated Brain Hospital of Nanjing Medical University (No. 2018-KY010-01, No. 2020-KY010-02, No.2021-KY029-01, No. 2021-KY009-01, No. 2022-KY042-01, No. ChiCTR2000034533, No. ChiCTR1900022287).

**S.2 Inclusion and Exclusion Criteria**

Inclusion criteria for SCD subjects were based on published SCD criteria, as proposed by the Subjective Cognitive Decline Initiative (SCD-I) [1], which we also described in our previously published article [2]. The inclusion criteria for our database is as follows: (a) self-reported persistent memory decline, which was confirmed by an informant; (b) a Subjective Cognitive Decline Questionnaire (SCD-Q) score > 5 [3-5]; (c) performance within the normal range on MMSE and MoCA (adjusted for age and education); (d) Clinical Dementia Rating (CDR) = 0; (e) subjects aged between 50 and 80 years old.

Inclusion criteria for aMCI subjects were as per the diagnostic criteria as defined by Peterson et al. [6, 7], the revised consensus standards presented by Winblad et al. [8], and the criteria as described in our preceding studies [2, 9, 10] which comprised of the following: (a) a memory complaint, preferably corroborated by an informant or the subject for more than 3 months; (b) objective memory impairment, adjusted for age and educational level; (c) normal general cognitive function of MMSE score equal or above 24; (d) none or minimal impairment in daily living activities; (e) CDR=0.5; (f) subjects aged between 50 and 80 years old; (g) absence of dementia symptoms that were not sufficient to meet the criteria of the National Institute of Neurological and Communicative Disorders and Stroke or the AD and Related Disorders Association criteria for AD.

Inclusion criteria for HCs were as follows: (a) no memory complaints; (b) normal cognitive performance matched for age and education; (c) CDR=0; (d) MMSE ≥ 26; and (e) subjects aged between 50 and 80 years old [2, 11].

All participants met the following exclusion criteria, as described in our earlier studies[2, 9, 10]: (a) a past history of stroke (modified Hachinski Ischemic Scale Score of > 4), alcoholism, head injury, brain tumors, Parkinson’s disease, epilepsy, encephalitis, major depression (excluded by HAMD), or other neurological or psychiatric illness, as evaluated by clinical assessment and case history; (b) any major medical illness (e.g., cancer, anemia, thyroid dysfunction, syphilis, or HIV); (c) severe visual or hearing loss; (d) inability to complete neuropsychological tests or with a contraindication for MRI; (e) T2-weighted MRI showing major changes in white matter (WM), infarction, or other lesions, as assessed by two experienced radiologists; and (f) no history or current use of psychotropic medications.

**S.3 Neuropsychological Assessments**

Neuropsychological assessments were performed as described in our previous research papers [2, 9, 11-13]. All subjects had a standardized clinical interview and underwent comprehensive neuropsychological assessments by 3 neuropsychologists (Dr. Xue, Qi, and Liu). The evaluation included the Mini Mental State Examination (MMSE), Montreal Cognitive Assessment (MoCA), Mattis Dementia Rating Scale (MDRS), Auditory Verbal Learning Test - immediate recall (AVLT-IM), Auditory Verbal Learning Test – 5-min delayed recall (AVLT-5-min-DR), Auditory Verbal Learning Test –20-min delayed recall (AVLT-20-min-DR), Logical Memory Test –immediate recall (LMT-IM), Logical Memory Test –20-min delayed recall (LMT-20-min-DR), Rey-Osterrieth Complex Figure Test –20-min delayed recall (ROCFT-20min-DR), Clock Drawing Test (CDT), Rey-Osterrieth Complex Figure Test (ROCFT), Verbal Fluency Test (VFT), Digit Span Test (DST), Digital Symbol Substitution Test (DSST), Trail-Making Tests A and B (TMT-A and B), Stroop Color and Word Test A, B, and C, and Semantic Similarity (Similarity) test. These tests were used to evaluate general cognitive function, episodic memory, information processing speed, executive function, and visuo-spatial function.

**S.4 Image acquisition**

The NBH-ADsnp-2 data acquisition process was also depicted in our former articles [2, 9, 10, 14]. All MRI data were acquired using a 3.0 Tesla Verio Siemens scanner with an 8-channel head-coil at the Affiliated Brain Hospital of Nanjing Medical University (Nanjing, China). Resting-state functional images were collected while participants were instructed to rest with their eyes open, not to fall asleep, and not to think of anything in particular. The gradient-echo echo-planar imaging (GRE-EPI) sequence included 240 volumes. The parameters were as follows: repetition time (TR) = 2000 ms, echo time (TE) = 30 ms，number of slices = 36, thickness = 4.0 mm, gap = 0 mm, matrix = 64×64, flip angle (FA) = 90°, field of view (FOV) = 220 mm×220 mm, acquisition bandwidth = 100 kHz, voxel size = 3.4×3.4×4 mm^3^. The imaging process took approximately 8 minutes.

High-resolution T1-weighted images were obtained by a 3D magnetization-prepared rapid gradient-echo (MPRAGE) sequence. The parameters were as follows: TR = 1900 ms, TE = 2.48 ms, inversion time (TI) = 900 ms, number of slices = 176, thickness = 1.0 mm, gap = 0.5 mm, matrix = 256×256, FA = 9°, FOV = 256 mm×256 mm, voxel size = 1×1×1 mm^3^. The imaging process lasted for approximately 4.26 minutes.

Moreover, routine axial T2-weighted images were acquired to rule out subjects with major changes in white matter (WM), cerebral infarction or other lesions using flair sequence as follows: TR = 8400 ms, TE = 94 ms, FA= 150°, acquisition matrix = 256×256, FOV = 230×230 mm, thickness = 5.0 mm, gap = 0 mm, and number of slices = 20. The imaging process took approximately 2.50 minutes to complete.

**S.5 Structural MRI Data Preprocessing**

The standard processing procedures included (1) correction of minor head movements; (2) correction of signal strength nonuniformities caused by magnetic field inhomogeneities; (3) removal of no-brain issue (skull stripping); (4) affine registration to the Talairach atlas and segmentation of the subcortical white matter and deep gray matter structures; (5) tessellation of the gray-to-white and gray-to-cerebrospinal fluid (CSF) surface boundaries; (6) automatic correction of topology defects; (7) surface deformation for optional placement of the gray-to-white and gray-to-CSF boundaries and smoothing with a 10 mm FWHM Gaussian smoothing kernel; (8) surface inflation and registration to a spherical atlas for inter subject matching of cortical folding patterns; (9) parcellation of the cortical mantle into the brain areas of the Desikan−Killiany atlas. All surface models were visually checked for inaccuracy and manually edited as needed.

1. Jessen F, Amariglio RE, van Boxtel M, Breteler M, Ceccaldi M, Chételat G, Dubois B, Dufouil C, Ellis KA, van der Flier WM, Glodzik L, van Harten AC, de Leon MJ, et al. A conceptual framework for research on subjective cognitive decline in preclinical Alzheimer's disease. Alzheimers Dement. 2014; 10:844–52.

<https://doi.org/10.1016/j.jalz.2014.01.001> PMID:[24798886](https://pubmed.ncbi.nlm.nih.gov/24798886)

2. Xue C, Yuan B, Yue Y, Xu J, Wang S, Wu M, Ji N, Zhou X, Zhao Y, Rao J, Yang W, Xiao C, Chen J. Distinct disruptive patterns of default mode subnetwork connectivity across the spectrum of preclinical Alzheimer’s disease. Front Aging Neurosci. 2019; 11:307.

<https://doi.org/10.3389/fnagi.2019.00307> PMID:[31798440](https://pubmed.ncbi.nlm.nih.gov/31798440)

3. Yan T, Wang W, Yang L, Chen K, Chen R, Han Y. Rich club disturbances of the human connectome from subjective cognitive decline to Alzheimer’s disease. Theranostics. 2018; 8:3237–55.

<https://doi.org/10.7150/thno.23772> PMID:[29930726](https://pubmed.ncbi.nlm.nih.gov/29930726)

4. Cedres N, Machado A, Molina Y, Diaz-Galvan P, Hernández-Cabrera JA, Barroso J, Westman E, Ferreira D. Subjective cognitive decline below and above the age of 60: a multivariate study on neuroimaging, cognitive, clinical, and demographic measures. J Alzheimers Dis. 2019; 68:295–309.

<https://doi.org/10.3233/JAD-180720> PMID:[30741680](https://pubmed.ncbi.nlm.nih.gov/30741680)

5. Hao L, Wang X, Zhang L, Xing Y, Guo Q, Hu X, Mu B, Chen Y, Chen G, Cao J, Zhi X, Liu J, Li X, et al. Prevalence, risk factors, and complaints screening tool exploration of subjective cognitive decline in a large cohort of the Chinese population. J Alzheimers Dis. 2017; 60:371–88.

<https://doi.org/10.3233/JAD-170347> PMID:[28869471](https://pubmed.ncbi.nlm.nih.gov/28869471)

6. Dunn CJ, Duffy SL, Hickie IB, Lagopoulos J, Lewis SJ, Naismith SL, Shine JM. Deficits in episodic memory retrieval reveal impaired default mode network connectivity in amnestic mild cognitive impairment. Neuroimage Clin. 2014; 4:473–80.

<https://doi.org/10.1016/j.nicl.2014.02.010> PMID:[24634833](https://pubmed.ncbi.nlm.nih.gov/24634833)

7. Petersen RC, Smith GE, Waring SC, Ivnik RJ, Tangalos EG, Kokmen E. Mild cognitive impairment: clinical characterization and outcome. Arch Neurol. 1999; 56:303–08.

<https://doi.org/10.1001/archneur.56.3.303> PMID:[10190820](https://pubmed.ncbi.nlm.nih.gov/10190820)

8. Winblad B, Palmer K, Kivipelto M, Jelic V, Fratiglioni L, Wahlund LO, Nordberg A, Bäckman L, Albert M, Almkvist O, Arai H, Basun H, Blennow K, et al. Mild cognitive impairment—beyond controversies, towards a consensus: report of the international working group on mild cognitive impairment. J Intern Med. 2004; 256:240–46.

<https://doi.org/10.1111/j.1365-2796.2004.01380.x> PMID:[15324367](https://pubmed.ncbi.nlm.nih.gov/15324367)

9. Chen J, Chen G, Shu H, Chen G, Ward BD, Wang Z, Liu D, Antuono PG, Li SJ, Zhang Z, and Alzheimer’s Disease Neuroimaging Initiative. Predicting progression from mild cognitive impairment to Alzheimer’s disease on an individual subject basis by applying the CARE index across different independent cohorts. Aging (Albany NY). 2019; 11:2185–201.

<https://doi.org/10.18632/aging.101883> PMID:[31078129](https://pubmed.ncbi.nlm.nih.gov/31078129)

10. Chen J, Shu H, Wang Z, Zhan Y, Liu D, Liao W, Xu L, Liu Y, Zhang Z. Convergent and divergent intranetwork and internetwork connectivity patterns in patients with remitted late-life depression and amnestic mild cognitive impairment. Cortex. 2016; 83:194–211.

<https://doi.org/10.1016/j.cortex.2016.08.001> PMID:[27570050](https://pubmed.ncbi.nlm.nih.gov/27570050)

11. Chen J, Shu H, Wang Z, Zhan Y, Liu D, Liu Y, Zhang Z. Intrinsic connectivity identifies the sensory-motor network as a main cross-network between remitted late-life depression- and amnestic mild cognitive impairment-targeted networks. Brain Imaging Behav. 2020; 14:1130–42.

<https://doi.org/10.1007/s11682-019-00098-4> PMID:[31011952](https://pubmed.ncbi.nlm.nih.gov/31011952)

12. Chen J, Shu H, Wang Z, Liu D, Shi Y, Zhang X, Zhang Z. The interaction of APOE genotype by age in amnestic mild cognitive impairment: a voxel-based morphometric study. J Alzheimers Dis. 2015; 43:657–68.

<https://doi.org/10.3233/JAD-141677> PMID:[25114090](https://pubmed.ncbi.nlm.nih.gov/25114090)

13. Chen J, Duan X, Shu H, Wang Z, Long Z, Liu D, Liao W, Shi Y, Chen H, Zhang Z. Differential contributions of subregions of medial temporal lobe to memory system in amnestic mild cognitive impairment: insights from fMRI study. Sci Rep. 2016; 6:26148.

<https://doi.org/10.1038/srep26148> PMID:[27184985](https://pubmed.ncbi.nlm.nih.gov/27184985)

14. Chen J, Yan Y, Gu L, Gao L, Zhang Z. Electrophysiological processes on motor imagery mediate the association between increased gray matter volume and cognition in amnestic mild cognitive impairment. Brain Topogr. 2020; 33:255–66.

<https://doi.org/10.1007/s10548-019-00742-8> PMID:[31691911](https://pubmed.ncbi.nlm.nih.gov/31691911)
